# SIPA1 boosts migration and proliferation, and blocks apoptosis of glioma by activating the phosphorylation of the FAK signaling pathway

**DOI:** 10.5937/jomb0-32903

**Published:** 2022-02-02

**Authors:** Yuan Du, Shenglan Li, Tong Zhou, Jing Zhao, Jiguang Liu

**Affiliations:** 1 Jiamusi University, School of Basic Medicine Science, Jiamusi, China; 2 Central Hospital of Jamusi City, Jiamusi, China; 3 Capital Medical University, Beijing Tiantan Hospital, Department of Neuro-oncology, Cancer center, Beijing, China; 4 Jiamusi University, School of Pharmacy, Jiamusi, China; 5 First Affiliated Hospital of Jiamusi University, Clinical Laboratory, Jiamusi, China; 6 Jiamusi University, School of Stomatology, Jiamusi, China

**Keywords:** SIPA1, FAK, phosphorylation, glioma, SIPA1, FAK, fosforilacija, gliom

## Abstract

**Background:**

We aimed to analyze the regulatory effects of SIPA1 (signal-induced proliferation-associated protein 1) on glioma progression and the dominant signaling pathway.

**Methods:**

Differential level of SIPA1 in glioma and normal tissues and cells was determined. Migratory, proliferative, apoptotic and cell cycle progression changes in A172 cells with overexpression or knockdown of SIPA1 were examined. Finally, protein levels of phosphorylated FAKs in A172 cells intervened by SIPA1, and the FAK inhibitor PF562271 were detected.

**Results:**

SIPA1 was upregulated in glioma cases. Knock-down of SIPA1 reduced migratory and proliferative rates of glioma cells, increased apoptotic cell rate, and declined cell ratio in the S phase. The knockdown of SIPA1 also downregulated cell cycle proteins. In addition, SIPA1 upregulated phosphorylated FAKs in A172 cells and thus boosted malignant phenotypes of glioma.

**Conclusions:**

SIPA1 is upregulated in glioma that boosts migratory and proliferative potentials of glioma cells by activating the phosphorylation of the FAK signaling pathway.

## Abstract (sr)

**Uvod:**

Cilj nam je bio da analiziramo regulatorne efekte SIPA1 na progresiju glioma i dominantni signalni put.

**Metode:**

Određen je diferencijalni nivo SIPA1 u gliomu i normalnim tkivima i ćelijama. Ispitivane su migratorne, proliferativne, apoptotičke i promene u progresiji ćelijskog ciklusa u ćelijama A172 sa prekomernom ekspresijom ili obaranjem SIPA1. Konačno, otkriveni su nivoi proteina fosforilisanih FAK u ćelijama A172 sa intervencijom SIPA1 i inhibitorom FAK PF-562271.

**Rezultati:**

SIPA1 je uvećan u slučajevima glioma. Pad SIPA1 je smanjio migracijsku i proliferativnu stopu ćelija glioma, povećao apoptotičku ćelijsku stopu i smanjio ćelijski odnos u S fazi. Snižavanjem SIPA1 takođe su sniženi i proteini ćelijskog ciklusa. SIPA1 je povećao fosforilisani FAK u ćelijama A172 i tako pojačao maligne fenotipe glioma.

**Zaključak:**

SIPA1 je povećan kod glioma i pojačava migratorne i proliferativne potencijale ćelija glioma aktiviranjem fosforilacije signalnog puta FAK.

## Introduction

Gliomas are the leading malignant tumors of the central nervous system that originate from glial cells of the neuroectoderm. They represent 80% of primary intracranial malignancies [1]. The average survival time of glioma is only 12-14 months [2]. Currently, surgery combined radiotherapy, chemotherapy and biotherapy is preferred to glioma patients, although they can only prolong the survival for months. The prognosis of glioma is extremely poor [3]. Clarification of the pathogenesis of glioma and the involvement of differentially expressed genes in glioma contributes to the improvement of clinical outcomes [4].

SIPA1 (signal-induced proliferation-associated protein 1) is a protein relevant to tumor invasiveness and metastasis. It is located on human chromosome 11q13.3, containing a zinc finger at the C terminal and a GTPase activator that is highly homologic with Rap1GAP at the N terminal [5] [6]. RapGAP protein constitutes Rap1GAP and SIPA1 [7]. As a specific RapGAP protein, SIPA1 negatively regulates Rap1 by converting it to the inactivate GDP-bound state, thus translocating signals into nuclei that mediates gene transcription [8]. Rap1 is highly homologic with Ras, sharing similar functions in regulating cell-cell connection, secretion, and adhesion [9]. In addition, SIPA1 also participates in the mediation of cell clonality, adhesion, and migration [10]. This study aims to explore the regulatory effects of SIPA1 on glioma and the dominant signaling pathway.

## Materials and Methods

### Collection of glioma samples

Glioma samples (n=32) were surgically resected and collected. Glioma cases were pathologically confirmed, and they did not have preoperative glioma treatments. Normal brain tissue samples (n=24) resected during craniocerebral decompression in patients with brain traumas were collected as controls. The Ethic Committee of The Central Hospital of Jamusi City approved this study, and written informed consent was obtained from each patient.

### Cell culture

The GBM-derived T98G and A172 cell lines, the grade III astrocytoma-derived U87 cell line and astrocyte cell line NHA (American Type Culture Collection (ATCC) (Manassas, VA, USA)) were cultivated in Dulbecco's Modified Eagle Medium (DMEM) (Gibco, Rockville, MD, USA) supplemented with 10% fetal bovine serum (FBS) (Gibco, Rockville, MD, USA) in an incubator containing 5% CO_2_ at 37°C. Cell passage was conducted at 80% of confluence.

### Cell transfection

Cells seeded in a 6-well plate were grown to 80% of confluence, followed by the transfection of vectors constructed by GenePharma (Shanghai, China) using Lipofectamine 2000 (Invitrogen, Carlsbad, CA, USA). Transfection efficacy was examined by quantitative real-time polymerase chain reaction (qRT-PCR) at 24 h.

### qRT-PCR

Cells were lysed in TRIzol (Invitrogen, Carlsbad, CA, USA) for 5 min, followed by incubation in 200 µL of chloroform. After 12,000×g centrifugation at 4°C for 5 min, the upper layer was collected and incubated with 500 µL of isopropanol. After 12,000×g centrifugation at 4°C for 10 min, the precipitant was washed in 1 mL of 75% ethanol and diluted in 20 µL of diethylpyrocarbonate (DEPC) water (Beyotime, Shanghai, China). RNA concentration was measured using NanoDrop 2000 (Thermo Fisher Scientific, Inc., Waltham, MA, USA). Using the PrimeScript™RT Master Mix, reversely transcribed complementary deoxyribose nucleic acids (cDNAs) were further subjected to qPCR.

SIPA1-Forward: 5’-TGCAAGATGGTGGCAGTCCTC-3’; SIPA1-Reverse: 5’-CTGCCCGCCTCCGACATGATC-3’; GAPDH-Forward: 5’-ACACCATGGGGAAGGTGAAG-3’; GAPDH-Reverse: 5’-GTGACCAGGCGCCCAATA-3’; Cyclin A2-Forward: 5’-CGCTGGCGGTACTGAAGTC-3’; Cyclin A2-Reverse: 5’-GAGGAACGGTGACATGCTCAT-3’; Cyclin D1-Forward: 5’-GCTGCGAAGTGGAAACCATC-3’; Cyclin D1-Reverse: 5’-CCTCCTTCTGCACACATTTGAA-3’; Cyclin E1-Forward: 5’-AAGGAGCGGGACACCATGA-3’; Cyclin E1-Reverse: 5’-ACGGTCACGTTTGCCTTCC-3’.

### Transwell

Cell suspension (5×10^4^/L) in serum-free medium and medium containing 10% FBS were respectively applied at the top and bottom of a Transwell insert pre-coated with 200 mg/mL Matrigel. After 24-h cell culture, cells migrated from the top to the bottom were fixed in 70% ethanol for 30 min and dyed in 0.2% crystal violet for 10 min, which were then observed and counted.

### 5-Ethynyl-2'-deoxyuridine (EdU)

Cell suspension (2×10^5^/L) was seeded in a 96-well plate and stained with EdU as recommended by the commercial kit (Beyotime, Shanghai, China). EdU-positive cells in 3 random fields per well were captured for calculating using Image J software (NIH, Bethesda, MD, USA).

### Flow cytometry

After 5-min centrifugation at 1,000 r/min and phosphate-buffered saline (PBS) washing twice, the precipitant was induced with 5 µL of Annexin V/FITC and 10 µL of Propidium Iodide (PI) in the dark for 15 min. Cell apoptosis was analyzed by detecting FL1 (488 nm wavelength) and FL2 gate (633 nm wavelength). In addition, cell cycle distribution was analyzed using the CellQuestTMD Analysis Software (BD Biosciences, Franklin Lakes, NJ, USA).

### Western blot

After 30-min lysis of cells, and 15-min centrifugation at 4°C, 12,000 rpm, protein samples were prepared for sodium dodecyl sulphate-polyacrylamide gel electrophoresis (SDS-PAGE) (30 µg per lane) and transfer on polyvinylidene fluoride (PVDF) membranes (Millipore, Billerica, MA, USA). After blocking non-specific antigens on membranes, they were induced with primary and secondary antibodies under indicated conditions. Protein signals were detected using Luminol substrate solution.

### Statistical analysis

Statistical Product and Service Solutions (SPSS) 22.0 (IBM, Armonk, NY, USA) was used for statistical processing. Data were expressed as x̄ ± s, and differences between groups were compared using the independent t-test. A significant difference was set at P<0.05.

## Results

### Upregulation of SIPA1 in glioma

**Figure 1:**
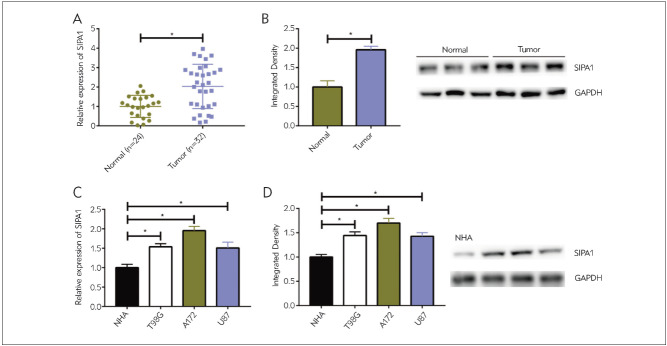
Upregulation of SIPA1 in glioma (A) The mRNA level of SIPA1 in glioma and normal brain tissues<br>(B) The protein level of SIPA1 in glioma and normal brain tissues<br>(C) The mRNA level of SIPA1 in glioma cell lines<br>(D) The protein level of SIPA1 in glioma cell lines<br>*P<0.05

Compared with normal brain tissues, mRNA and protein levels of SIPA1 were remarkably upregulated in glioma (Figure 1A, Figure 1B). Meanwhile, SIPA1 was more highly expressed in glioma cell lines than astrocytes (Figure 1C, Figure 1D). A172 cells were used for the following experiments since they expressed a relatively high abundance of SIPA1 in the three tested glioma cell lines.

### Knockdown of SIPA1 suppressed migratory and proliferative potentials of glioma

**Figure 2:**
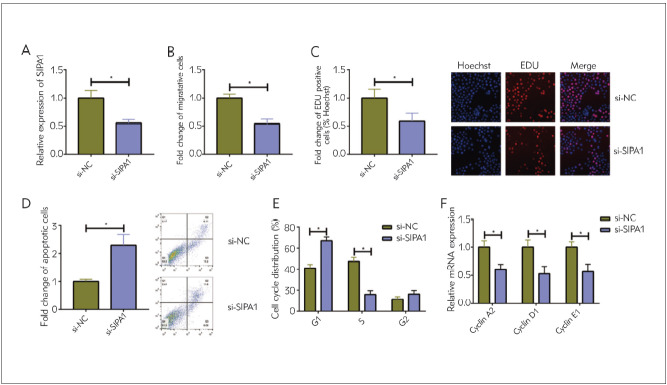
Knockdown of SIPA1 suppressed migratory and proliferative potentials of glioma (A) Transfection efficacy of si-SIPA1 in A172 cells<br>(B) Migration in A172 cells with SIPA1 knockdown<br>(C) EdU-positive ratio in A172 cells with SIPA1 knockdown (magnification = 40×)<br>(D) Apoptosis in A172 cells with SIPA1 knockdown<br>(E) Cell cycle distribution in A172 cells with SIPA1 knockdown<br>(F) Relative levels of Cyclin A2, Cyclin D1 and Cyclin E1 in A172 cells with SIPA1 knockdown<br>*P<0.05

SIPA1 level was effectively suppressed by transfection of si-SIPA1 in A172 cells (Figure 2A). After the knockdown of SIPA1, the migratory cell number (Figure 2B) and EdU-positive ratio (Figure 2C) were markedly reduced. In addition, cell apoptosis was stimulated by transfection of si-SIPA1 (Figure 2D). Flow cytometry data showed that the knockdown of SIPA1 in A172 cells arrested cell cycle progression in the G1 phase, which was further supported by the downregulation of cell cycle proteins Cyclin A2, Cyclin D1 and Cyclin E1 in glioma cells with SIPA1 knockdown (Figure 2E, Figure 2F).

### Overexpression of SIPA1 boosted migratory and proliferative potentials of glioma

**Figure 3:**
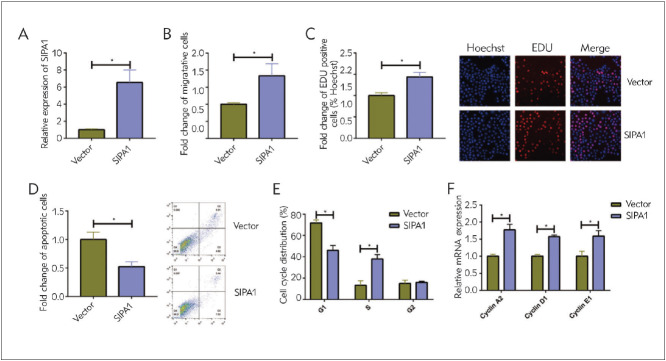
Overexpression of SIPA1 suppressed migratory and proliferative potentials of glioma (A) Transfection efficacy of SIPA1 overexpression vector in A172 cells<br>(B) Migration in A172 cells with SIPA1 overexpression<br>(C) EdU-positive ratio in A172 cells with SIPA1 overexpression (magnification = 40×)<br>(D) Apoptosis in A172 cells with SIPA1 overexpression<br>(E) Cell cycle distribution in A172 cells with SIPA1 overexpression<br>(F) Relative levels of Cyclin A2, Cyclin D1 and Cyclin E1 in A172 cells with SIPA1 overexpression<br>*P<0.05

We analyzed phenotype changes of A172 cells overexpressing SIPA1 further (Figure 3A). Overexpression of SIPA1 markedly enhanced migratory and proliferative potentials of glioma cells (Figure 3B, Figure 3C). In addition, the apoptosis rate was reduced in A172 cells overexpressing SIPA1 (Figure 3D). Besides, overexpression of SIPA1 remarkably prolonged the S phase and upregulated Cyclin A2, Cyclin D1 and Cyclin E1 (Figure 3E, Figure 3F).

### Overexpression of SIPA1 activated phosphorylation of FAK

**Figure 4:**
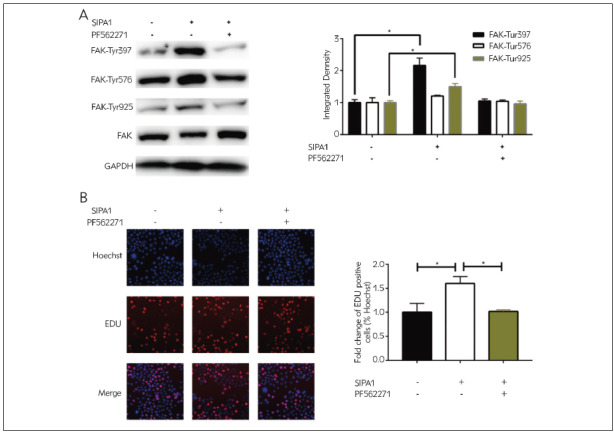
Overexpression of SIPA1 activated phosphorylation of FAK. A172 cells were transfected with negative control or SIPA1 overexpression vector, followed by either treatment of PF-562271 or not (A) Protein levels of Phospho-FAK (Try397), Phospho-FAK (Try576), Phospho-FAK (Try925) and FAK<br>(B) EdU-positive ratio in A172 cells (magnification = 40×)<br>*P<0.05

Interestingly, overexpression of SIPA1 in A172 cells upregulated Phospho-FAK (Try397), Phospho-FAK (Try576) and Phospho-FAK (Try925), which were reversed by treatment of the FAK inhibitor PF-562271 (Figure 4A). To further explore the involvement of the phosphorylated FAK in SIPA1-induced glioma progression, proliferative ability in glioma cells overexpressing SIPA1 intervened by PF-562271 was examined. As expected, the intervention of PF-562271 reduced the EdU-positive rate, indicating that the phosphorylation of FAK did participate in glioma progression boosted by SIPA1 (Figure 4B).

## Discussion

Glioma is a complicated malignant tumor. Its pathogenesis remains largely unclear, and brain traumas, nitrite food, occupational hazard and radiation exposure may be potential risk factors of glioma. Besides, immune factors are closely associated with the development of glioma, involving Treg, CD3^+^T, CD4^+^T and CD8^+^T cells [11]. Therefore, differentially expressed genes in gliomas have been well concerned. They can be utilized as specific biomarkers for guiding the screening, diagnosis and treatment, and predicting the prognosis of glioma [12]. The development of targeted therapy based on these biomarkers is a promising approach to improving glioma patients' poor prognosis [13] [14].

The cancer-associated role of SIPA1 differs in human malignant tumors. Hunter et al. [15] first identified that SIPA1 is located on the metastasis efficiency modifier locus mtes1. SIPA1 SNP remarkably influences the function of Rap GTPase. They demonstrated that intervention of SIPA1 in nude mice markedly alters the metastatic ability of cancer cells. Minato et al. [16] suggested that SIPA1 prevents cell adhesion to fibronectin by inhibiting Rap1GTP in Hela cells. Through mediating the interaction between SIPA1 and Rap1GTP, AF-6 inhibits integrininduced cell adhesion [17]. The diasporin pathway can regulate transcription of extracellular matrix (ECM) genes, pTEN and Trp53, which is a tumor progression-associated pathway regulated by SIPA1 [18] [19]. In hematological malignancies, SIPA1 acts as a tumor-suppressor gene. SIPA1 knockout mice showed T cell non-responsiveness before bone marrow dysfunction, which eventually leads to the development of delayed myeloid leukemia [20]. SIPA1 is distributed in nuclei of breast cancer cell line MDA-MB-231. By promoting the expression of integrin 1 by acting on its promoter, SIPA1 further activates the phosphorylation of FAK, and thus regulates invasiveness and morphology of breast cancer cells through the MMP9 signaling and F-actin, respectively [21]. *In vivo* overexpression of SIPA1 enhances tumorigenesis of prostate cancer in SCID mice by inhibiting the binding between collagen and fibronectin, thus inactivating ECM-induced activation of Rap1. Meanwhile, overexpression of SIPA1 downregulates Brd4, which further attenuates the binding between prostate cancer cells and ECM [22]. The regulatory effect of SIPA1 on the migratory capacity of colorectal carcinoma (CRC) is quite the opposite of that in breast cancer and prostate cancer. A clinical trial involving 94 CRC patients revealed that the relative level of SIPA1 is negatively correlated to metastatic lymphatic rate. Knockdown of SIPA1 markedly triggers the migratory ability of CRC cells [23]. In the present study, SIPA1 was highly expressed in glioma cases, which boosted migratory and proliferative capacities of glioma cells and inhibited cell apoptosis.

FAK is overactivated or over-phosphorylated in multiple types of cancer cells, leading to malignant migration, proliferation, adhesion and EMT [24]. In addition to the kinase function, FAK also serves as a scaffold for protein complexes that regulates cancer development [25]. Here, overexpression of SIPA1 upregulated Phospho-FAK (Try397), Phospho-FAK (Try576) and Phospho-FAK (Try925), which were reversed by treatment of the FAK inhibitor PF-562271. The treatment of PF-56271 abolished the boosted proliferative ability of glioma by overexpression of SIPA1. It is indicated that the phosphorylation of FAK was involved in the glioma progression boosted by SIPA1.

There were limitations in the present study. First of all, the sample size of glioma and normal brain tissues was limited. Therefore, the clinical significance of SIPA1 in glioma needs to be further validated in more samples. Secondly, *in vivo* experiments are lneeded to verify the tumorigenic role of SIPA1 in glioma.

## Conclusion

SIPA1 is upregulated in glioma, which boosts malignant progression of glioma by activating the phosphorylation of the FAK signaling pathway.

## Dodatak

### Financial Disclosure

The authors declared that this study had received no financial support.

### Conflict of interest statement

All the authors declare that they have no conflict of interest in this work.
